# Psychosocial determinants of healthcare use costs in kidney transplant recipients

**DOI:** 10.3389/fpubh.2023.1158387

**Published:** 2023-06-02

**Authors:** Luigi Zerbinati, Franco Guerzoni, Nicola Napoli, Antonio Preti, Pasquale Esposito, Rosangela Caruso, Francesca Bulighin, Alda Storari, Luigi Grassi, Yuri Battaglia

**Affiliations:** ^1^Department of Neuroscience and Rehabilitation, Institute of Psychiatry, University of Ferrara, Ferrara, Italy; ^2^Programming and Management Control Service, Unit of Controls, St. Anna University-Hospital, Ferrara, Italy; ^3^Programming and Management Control Service, Statistics Service, St. Anna University-Hospital, Ferrara, Italy; ^4^Department of Neuroscience, University of Turin, Turin, Italy; ^5^Division of Nephrology, Dialysis and Transplantation, Department of Internal Medicine, University of Genoa and IRCCS Ospedale Policlinico San Martino, Genoa, Italy; ^6^Department of Medicine, University of Verona, Verona, Italy; ^7^Nephrology and Dialysis Unit, St. Anna University-Hospital, Ferrara, Italy; ^8^Nephrology and Dialysis Unit, Pederzoli Hospital, Verona, Italy

**Keywords:** psychiatric diagnosis, ICD, DCPR, mood, somatization, distress, hospital admission, emergency access

## Abstract

**Introduction:**

Psychosocial factors frequently occur in kidney transplant recipients (KTRs), leading to behavioral alterations and reduced therapeutic adherence. However, the burden of psychosocial disorders on costs for KTRs is unknown. The aim of the study is to identify predictors of healthcare costs due to hospital admissions and emergency department access in KTRs.

**Methods:**

This is a longitudinal observational study conducted on KTRs aged >18 years, excluding patients with an insufficient level of autonomy and cognitive disorder. KTRs underwent psychosocial assessment via two interviews, namely the Mini-International Neuropsychiatric Interview 6.0 (MINI 6.0) and the Diagnostic Criteria for Psychosomatic Research Interview (DCPR) and via the Edmonton Symptom Assessment System Revised (ESAS-R) scale, a self-administrated questionnaire. Sociodemographic data and healthcare costs for hospital admissions and emergency department access were collected in the 2016–2021 period. Psychosocial determinants were as follows: (1) ESAS-R psychological and physical score; (2) symptomatic clusters determined by DCPR (illness behavior cluster, somatization cluster, and personological cluster); and (3) ICD diagnosis of adjustment disorder, anxiety disorder, and mood disorder. A multivariate regression model was used to test the association between psychosocial determinants and total healthcare costs.

**Results:**

A total of 134 KTRs were enrolled, of whom 90 (67%) were men with a mean age of 56 years. A preliminary analysis of healthcare costs highlighted that higher healthcare costs are correlated with worse outcomes and death (*p* < 0.001). Somatization clusters (*p* = 0.020) and mood disorder (*p* < 0.001) were positively associated with costs due to total healthcare costs.

**Conclusions:**

This study showed somatization and mood disorders could predict costs for hospital admissions and emergency department access and be possible risk factors for poor outcomes, including death, in KTRs.

## 1. Introduction

Kidney transplantation (KT) is the most desired therapy for stage 5 chronic kidney disease as for these patients it represents the most cost-effective treatment, improving quality of life and prolonging survival (1, 2). In spite of being less costly than dialysis (3, 4), KT is however related to substantial costs (5–8), which can also derive not only from health problems such as cardiovascular disease, infections, graft rejections, and neoplastic disease (9–11) but also from the indirect effects of psychological conditions, such as depression or anxiety (12).

KT is often accompanied by high patient expectations, but it is indeed a stressful condition both physically and mentally that requires special adaptations encompassing changes in a patient's personal and financial life, meeting possibly unrealistic expectations, the possibility of rehospitalizations, infections, graft rejections, and the necessity of long-term immunosuppression therapy (13, 14). Indeed, 25 to 40% of KT recipients (KTRs) have been found to develop mood and anxiety disorders in the post-transplant period (15–21) according to the traditional Diagnostic and Statistical Manual of Mental Disorders (DSM) or the International Classification of Diseases (ICD). Furthermore, 60% of KTRs have shown some form of psychological distress when using the Diagnostic Criteria for Psychosomatic Research (DCPR) (22), a diagnostic and conceptual framework whose aim is to capture psychological dimensions and subthreshold syndromes (23, 24). These conditions are particularly relevant as they may generate dysfunctional illness behaviors (e.g., somatization, frequent attender behavior, and illness denial) that are associated with worse outcomes (12), medical non-adherence (25, 26), decreased quality of life, and increased costs (27, 28). More importantly, psychological conditions are both identifiable and treatable (22, 29–33), thus representing additional superfluous costs for the healthcare systems.

While many studies have highlighted the detrimental effects of psychosocial conditions on KTRs, this is the first study with the intent to directly investigate the contribution of psychiatric and psychosocial diagnoses as identified by both ICD and DCPR systems on healthcare use costs in KTRs. Specifically, using linear regression models, we aimed to identify predictors of total healthcare costs due to hospital admissions and emergency department access.

## 2. Methods

A monocenter prospective observational longitudinal study was performed at the kidney transplant center of the Ferrara University Hospital from 2016 to 2021. The study was conducted according to the 1995 Declaration of Helsinki and its revisions (34). The Ethical Committee of the local academic hospital approved the protocol of the study (Protocol n: 151297, 2016). All participants signed written informed consent.

Inclusion criteria were age ≥ 18 years and being a recipient of a kidney from a cadaveric or living donor. Exclusion criteria were an insufficient level of autonomy (Karnofsky Performance Status Scale <50) and the presence of cognitive disorders (Mini-Mental State Examination <24). Two individual interviews, namely the Mini-International Neuropsychiatric Interview (MINI6.0) (35) and the Diagnostic Criteria for Psychosomatic Research Semi-Structured Interview (36) were administered by the same psychiatrist, an expert in psychosomatic research (L.Z.). A self-reporting instrument, the Edmonton Symptom Assessment System revised (ESAS-Revised) in the Italian language, was also filled in by patients. The characteristics of the above tools were extensively described elsewhere (37, 38). Briefly, the MINI6.0 is a structured diagnostic interview for assessing the major psychiatric disorders in ICD-10, which was used to make a psychiatric diagnosis. A DCPR semi-structured interview evaluates the presence of 12 syndromes divided into three different clusters: (1) abnormal illness behavior (AIB) (i.e., Disease Phobia, Health Anxiety, Illness Denial, and Thanatophobia); (2) somatization (i.e., Functional Somatic Symptoms Secondary to a Psychiatric Disorder, Persistent Somatization, Conversion Symptoms, and Anniversary Reaction); and (3) personological and psychological dimensions frequently diagnosable in KTRs (39) (i.e., Alexithymia, Type A Behavior, Irritable Mood, and Demoralization).

The ESAS-R is a pragmatic patient-centered symptom assessment tool with a visual analog scale, designed to assist in the assessment of six physical (i.e., pain, tiredness, nausea, drowsiness, lack of appetite, and shortness of breath) and four psychological (i.e., depression, anxiety, feeling of not being well, and emotional distress) symptoms. In particular, the physical symptoms are assessed objectively (i.e., pain is based on a knowledge of pain behaviors; shortness of breath as accelerated respirations causing patient distress; tiredness as lack of energy; lack of appetite, nausea, and drowsiness as the presence of eating, retching/vomiting, and sleep, respectively). The items can be summed in order to create subscales of psychological, physical, and total distress, which can be used to monitor symptoms and screen for mental and psychological disorders. It has been validated in dialysis patients (40) and kidney transplant cohorts (41). The Italian version shows an acceptable level of validity and good psychometric properties in KTRs (33, 42).

All data, including clinical characteristics and routine biochemistry, were collected from digital patients' archives.

The following variables were used as a measure of outcome: total healthcare costs due to hospital admissions in the 2016–2021 period; and total healthcare costs due to emergency department access in the 2016–2021 period. Costs, covered by Italy's National Health Service, were expressed in euros (€), the Italian currency, and were extracted from a hospital software database, searching for each patient record both the type of medical service delivered and the related amount charged across the period from 2016 to 2021.

As predictors of healthcare costs, we used the following psychosocial determinants, all measured before the outcome: ESAS, as a severity measure of physical and physiological symptoms; symptomatic clusters as measured by the DCPR; and clinical diagnosis according to MINI6.0 within the mood, anxiety, and adjustment disorder spectrum. Age (years), sex (men versus women), body mass index (BMI; kg/m^2^), time under dialysis before the transplant (months), transplant vintage (months), estimated glomerular filtration rate (eGFR) (ml/min), blood creatinine (mg/dl), blood albumin (g/dl), blood hemoglobin (g/dl), blood phosphate (mg/dl), blood calcium (mmol/l), blood inactive vitamin D (ng/ml), and past psychopathology (positive history versus no history) were entered as covariates in the analysis to control for variables that can affect healthcare use or somatic outcomes of the kidney transplant.

### 2.1. Statistical analysis

Data were entered in Excel, then coded and analyzed using the Statistical Package for Social Sciences (SPSS) version 28. All tests were two-tailed, with alpha set at *p* < 0.05.

Descriptive statistics were reported as means with standard deviation and range, or as counts and percentages. A regression model to test the association of our predictors to the outcomes was used. A preliminary exploration of the association of each variable with the outcomes, using univariate linear regression models, was performed. Afterward, a stepwise multivariable regression model to evaluate the association of our predictors with the outcomes were tested, by taking into account the considered covariates with the significance level for removal fixed at *p* < 0.10. In the model, discrete variables were entered as continuous values while nominal variables were entered as dichotomous [absent (0) vs. present (1)] values.

The minimum required sample size for multiple regression, given a desired power of 80% at alpha = 0.05 with 21 predictors and aiming at detecting an effect size of *f*^2^ = 0.20, was 124 participants. The calculation was carried out according to Soper (43). Multicollinearity was measured with the variance inflation factor (VIF), using a cut-off of 2.5 as a threshold to consider the presence of multicollinearity that could affect the regression model (44). As an effect size of the linear regression model, we used Cohen's *f*^2^, according to the formula: *f*^2^ = R^2^/(1–R^2^). By convention (45), *f*^2^ effect sizes of 0.02, 0.15, and 0.35 are considered small, medium, and large, respectively.

## 3. Results

Overall, 134 kidney transplant recipients, of whom only 10 were from living donors, were included in the longitudinal study, of which 90 (67%) were men and 44 (33%) were women. Nine patients declined to participate (six for work or family reasons and three because of health reasons). Men and women did not differ in demographic characteristics, biochemical values, and healthcare costs except for eGFR, blood creatinine, and personological cluster (Table 1). In fact, eGFR was on average higher in women (76.8 ± 27.2) than in men (58.6 ± 21.02): *t* = 3.90; *p* < 0.001. Conversely, blood creatinine was on average higher in men (1.5 ± 0.5) than in women (1.2 ± 0.4): *t* = −3.49; *p* < 0.001. Men [n = 51 (57%)] were more likely than women (n = 15 (34%)] to have a personological cluster (χ^2^ = 5.15; df = 1; *p* = 0.023). Above all, kidney transplant patients were Caucasians, coming from local districts.

**Table 1:** Distribution in the sample (*n* = 134) of the variables included in the study according to sex.

**Clinical and biochemical variables**	**Males (*n =* 90)**	**Females** **(*n =* 44)**	**Statistics**	**Effect size**
Age (years)^*^	55.2 (11.7)	58.0 (12.6)	*t =* 1.29; *p =* 0.199	Hedges'g = 0.23
BMI (m^2^/kg)^*^	24.5 (3.2)	24.5 (4.0)	*t =* 0.0; *p =* 1.00	Hedges'g = 0.00
Time under dialysis (months)^*^	30.8 (30.8)	26.6 (25.9)	*t =* 0.77; *p =* 0.44	Hedges'g = 0.14
Kidney graft vintage (months)^*^	116.9 (92.0)	125.7 (129.6)	*t =* 0.45; *p =* 0.65	Hedges'g = 0.08
Basal glomerular filtration rate (ml/min)^*^	58.6 (21.02)	76.8 (27.2)	*t =* 3.90; *p <* 0.001	Hedges'g = 0.78
Blood creatinine (mg/ml)^*^	1.5 (0.5)	1.2 (0.4)	*t =*−3.49; *p <* 0.001	Hedges'g = 0.64
Blood albumin (g/dl)^*^	58.2 (4.7)	57-9 (5.0)	*t =* 0.25; *p =* 0.801	Hedges'g = 0.04
Blood hemoglobin (g/dl)^*^	12.6 (1.6)	12.0 (1.3)	*t =* 2.21; *p =* 0.029	Hedges'g = 0.40
Blood phosphate (mg/dl)^*^	3.2 (0.6)	3.3 (0.8)	*t =* 0.76; *p =* 0.448	Hedges'g = 0.14
Blood calcium (mmol/l)^*^	2.5 (1.1)	2.6 (1.4)	*t =* 0.49; *p =* 0.620	Hedges'g = 0.09
Blood vitamin D (ng/ml)^*^	30.1 (11.6)	27.0 (10.4)	*t =* 1.48; *p =* 0.141	Hedges'g = 0.27
Past psychopathology, *n* (%)	27 (30.0)	14 (31.8)	χ^2^ = 0.0; df = 1; *p =* 0.988	Cramer's V = 0.02
**ESAS-R**				
ESAS psychological (scale score)^*^	10.4 (8.0)	11.4 (8.7)	*t =* 0.68; *p =* 0.498	Hedges'g = 0.12
ESAS physical (scale score)^*^	9.7 (7.7)	11.2 (9.7)	*t =* 0.944; *p =* 0.347	Hedges'g = 0.17
**DCPR diagnosis**				
Illness behavior cluster *n* (%)	23 (25)	12 (27)	χ^2^ = 0.0; df = 1; *p =* 0.988	Cramer's V = 0.02
Somatization cluster *n* (%)	10 (11)	9 (20)	χ^2^ = 1.42; df = 1; *p =* 0.233	Cramer's V = 0.13
Personological cluster *n* (%)	51 (57)	15 (34)	χ^2^ = 5.15; df = 1; *p =* 0.023	Cramer's V = 0.21
**ICD diagnosis**				
Adjustment disorder diagnosis, *n* (%)	14 (15)	7 (16)	χ^2^ = 0.0; df = 1; *p =* 1.00	Cramer's V = 0.005
Anxiety disorder diagnosis, *n* (%)	8 (9)	6 (13)	χ^2^ = 0.29;df = 1; *p =* 0.587	Cramer's V = 0.07
Mood disorder diagnosis, *n* (%)	7 (8)	4 (9)	χ^2^ = 0.0; df = 1; *p =* 1.00	Cramer's V = 0.02
**Healthcare Costs**				
Total healthcare costs due to hospital admissions and emergency department access (€)^*^	9077.63 (11020.19)	11448.00 (16602.49)	*t =* −0.984; *p =* 0.327	Hedges'g = 0.18

* Data are expressed as mean (standard deviation); BMI, body mass index; DCPR, Diagnostic Criteria for Psychosomatic Research; ESAS, Edmonton Symptom Assessment System; ICD, International Classification of Diseases. The effect size was reported for each comparison: Hedges' g was used for continuous variables and Cramer's V for categorical variables. The following thresholds were used: <0.20 = negligible; 0.20 to 0.50 = small; 0.50 to 0.80 = moderate; >0.80 = large for Hedges' g; <0.20 = small; 0.20 to 0.60 = moderate; >0.60 = large for Cramer's V.

At the end of the 2016–2021 observational period, the sample included 28 participants (21%) who had died. In preliminary investigations, increasing costs were related to an increased chance of a worse outcome, such as death (Table 2). Hence, healthcare use costs, either for hospital admission or emergency department access, represented an indicator of pejorative trajectories after a kidney transplant; in other words, higher costs were associated with poorer health.

**Table 2:** Outcome of kidney transplant according to health care use costs in the 2015–2021 observational period.

	**Alive at 2021 *n =* 106 (79%)**	**Dead at 2021** ***n =* 28 (21%)**	**Mean difference (95%CI)**
Total healthcare costs due to hospital admissions and emergency department access (€)^*^	6780.55 € (10287.60 €)	214498.91 € (16005.13 €)	14718.36 € (19630.47 € – 9806.24 €)

* ANOVA: F_[1;132]_ = 35.13; p <0.001.

The results of the univariate linear regression model are reported in Table 3. The beta can be interpreted as the increase (or decrease) in the outcome for each score point of a discrete variable or the presence of a nominal variable. For example, each year of age imports an increase of €216.61 in the total healthcare cost for hospital admissions. Hence, older people had higher healthcare use for hospital admissions, and as higher total healthcare costs for hospital admissions were related to a higher risk of death, they were also exposed to a greater risk of death. The standardized beta describes the strength of the association between the predictor and the outcome, and it is measured in units of standard deviation. The role of age was non-negligible as the change of 1 standard deviation in its value corresponded to a 19.9% of standard deviation in the dependent variable. Overall, only a minority of the predictors were related to the outcomes in a statistically significant manner. The presence of a mood disorder had the greatest impact on the outcomes and was associated with the largest increase in healthcare costs for both hospital admissions and emergency department access. It also had the largest association with the outcomes.

**Table 3:** Factors associated with total healthcare costs due to hospital admissions and emergency department access in kidney transplant recipients in a univariate linear regression.

	**Unstandardized Beta**	**Unstandardized standard error**	**Standard beta**	***t***	***p-*value**	**95%CI**
Sex (males)	−2370.58	2401.23	−0.085	−0.984	0.327	−7138.26 to 2397.10
Age (years)	216.61	92.96	0.199	2.330	0.021^*^	32.73 to 400.49
BMI (m^2^/kg)	−157.05	324.98	−0.042	−0.483	0.630	−799,71 to 485,60
Time under dialysis (months)	76.69	38.70	0.171	1.982	0.050^*^	0.14 to 153.24
Transplant vintage (months)	23.91	10.61	0.192	2.253	0.026^*^	2.92 to 44.89
Estimated glomerular filtration rate (ml/min)	−99.17	45.39	−0.187	−2.185	0.031^*^	−188.96 to −9.38
Blood creatinine (mg/ml)	5332.61	2055.03	0.220	2.595	0.011^*^	1267.56 to 9397.67
Blood albumin (g/dl)	−441.94	234.59	−0.162	−1.884	0.062	−905.98 to 22.10
Blood hemoglobin (g/dl)	−2026.10	719.67	−0.238	−2.815	0.006^*^	−3449.69 to −602.51
Blood phosphate (mg/dl)	2429.32	1727.04	0.122	1.407	0.162	−986.93 to 5845.58
Blood calcium (mmol/l)	152.92	966.00	0.014	0.158	0.874	−1757.92 to 2063.76
Blood vitamin D (ng/ml)	−61.10	101.00	−0.053	−0.605	0.546	−260.90 to 138.69
Past psychopathology (present)	3051.77	2450.89	0.108	1.245	0.215	−1796.33 to 7899.87
**ESAS**						
ESAS psychological (scale score)	340.93	135.25	0.214	2.521	0.013	73.40 to 608.47
ESAS physical (scale score)	298.69	133.15	0.192	2.243	0.027^*^	35.29 to 562.09
**DCPR diagnosis**						
Illness behavior cluster (present)	−475.99	2585.74	−0.016	−0.184	0.854	−5590.84 to 4638.86
Somatization cluster (present)	7149.41	3196.61	0.191	2.237	0.027^*^	826.20 to 13472.63
Personological cluster (present)	2343.51	2263.13	0.090	1.036	0.302	−2133.18 to 6820.21
**ICD diagnosis**						
Adjustment disorder diagnosis (present)	−3252.34	3112.10	−0.091	−1.045	0.298	−9408.39 to 2903.72
Anxiety disorder diagnosis (present)	575.72	3713.63	0.013	0.155	0.877	−6770.20 to 7921.64
Mood disorder diagnosis (present)	15372.52	3916.24	0.323	3.925	<0.001^*^	7625.80 to 23119.24

BMI, body mass index; DCPR, Diagnostic Criteria for Psychosomatic Research; ESAS, Edmonton Symptom Assessment System; ICD, International Classification of Diseases; s.e., standard error; std., standardized; VIF, variance inflation factor. ^*^Statistically significant.

We then proceeded to apply the stepwise multivariable model to evaluate the independent contribution of each predictor taking into account the covariates and the impact of the other predictors.

The model, concerning total healthcare costs due to hospital admissions and emergency department visits in the 2016-2021 period, extracted four variables as predictors of the outcome according to the predefined threshold for removal, with the other variables excluded for their negligible contribution (Table 4).

**Table 4:** Factors associated with total healthcare costs due to hospital admissions and emergency department access in kidney transplant recipients in a stepwise multivariable linear regression model.

	**UB**	**USE**	**SB**	***t***	***p*-value**	**95% CI**	**VIF**
Mood disorder diagnosis (present)	14214.12	3795.74	0.299	3.745	<0.001^*^	6703.61 to 21724.63	1.014
Somatization cluster (present)	6063.72	2976.39	0.162	2.037	0.044^*^	174.41 to 11953.02	1.007
Blood creatinine (mg/ml)	3949.73	1939.14	0.163	2.037	0.044^*^	112.81 to 7786.64	1.022
Transplant vintage (months)	21.27	9.89	0.171	2.151	0.033^*^	1.70 to 40.83	1.008

CI, Confidence interval; SB, Standard beta; UB, Unstandardized Beta; USE, Unstandardized standard error; VIF, variance inflation factor. ^*^Statistically significant.

In this model, the presence of a mood disorder diagnosis, the presence of the somatization cluster, transplant vintage, and blood creatinine were associated with higher healthcare costs due to hospital use [*F*_(4;128)_ = 7.88; *p* < 0.001; *R*^2^ = 19.6%; adjusted *R*^2^ = 17.1%; *f*^2^ = 0.244). Effect size, according to Cohen's *f*^2^, was estimated as medium to large. None of the variables in the model had a VIF higher than the suggested cutoff for multicollinearity.

Some diagnostic plots were used for testing the assumptions underlying the linear regression model by taking into account residual errors and fitted values. The model, which focused on total healthcare costs due to hospital use, showed a reasonable adaptation. In the residual vs. fitted plot, the residuals were spread equally around a horizontal line without distinct patterns (and the red line was approximately horizontal near zero), indicating a linear relationship. In the Q-Q plot, the majority of the residuals follow the straight dashed line. In the Scale-Location plot, there was a minor deviation from the homoscedasticity, confirmed by the Breusch–Pagan test (46): BP = 10.22, df = 4, *p* = 0.037. Just one point (case 19) was identified as influential based on Cook's distance (Supplementary Figure 1). We repeated the analysis by excluding this influential point (Table 5). In the new model, just the presence of a mood disorder diagnosis remained statistically related to healthcare costs due to hospital use, with a decrease in the overall effect size: *F*_(4;127)_ = 3.06; *p* = 0.019; *R*^2^ = 8.7%; adjusted *R*^2^ = 5.9%; *f*^2^ = 0.095. According to the diagnostic plots, the model had a good fit, there was no influential point according to Cook's distance, and there was no more deviation from the homoscedasticity (BP = 2.53, df = 4, *p* = 0.639).

**Table 5:** Factors associated with total healthcare costs due to hospital admissions and emergency department access in kidney transplant recipients in a stepwise multivariable linear regression model after the exclusion of the influential point.

	**UB**	**USE**	**SB**	***t***	***p*-value**	**95% CI**	**VIF**
Mood disorder diagnosis (present)	9312.50	3666.12	0.216	2.540	0.012^*^	2058.45 to 16566.54	1.013
Somatization cluster (present)	3430.03	2810.10	0.103	1.221	0.224	−2130.22 to 8990.24	1.002
Blood creatinine (mg/ml)	3006.24	1805.77	0.141	1.665	0.098	−566.78 to 6579.27	1.008
Transplant vintage (months)	10.20	9.45	0.092	1.079	0.282	−8.50 to 28.90	1.008

CI, Confidence interval; SB, standard beta; UB, unstandardized Beta; USE, unstandardized standard error; VIF, variance inflation factor. ^*^Statistically significant.

## 4. Discussion

In this study, we found that some psychosocial variables and clinical dimensions influenced total healthcare costs, hence total healthcare use, in kidney transplant recipients. In particular, a propensity to somatization and the presence of a mood disorder increased healthcare use costs for emergency department visits and hospital admissions. Furthermore, greater access to the emergency department and a higher chance of admission to the hospital were related to a greater risk of death in KTRs. These findings underline the need to assess psychosocial dimensions, such as somatization and mood disorder as predictors of healthcare use in kidney transplant recipients and possible risk factors for poor outcomes until death, using the DCPR semi-structured interview and MINI6.0 structured interview, respectively.

Mood disorders were also shown to increase total healthcare costs due to emergency department access and hospital admission. Regarding the former, this is in line with the literature, as approximately 50% of frequent emergency department users have a mental health diagnosis (47) and patients with mood disorders have been found to carry a 3-fold risk of frequent emergency department use (48). Besides a possible increase in emergency department use, higher costs might also be the result of the harmful effect of the mood disorder itself, thus raising the total healthcare costs due to hospital admission. Depression, which represents the most common type of mood disorder, specifically represents a risk factor for graft failure and post-transplant mortality (12), and it is associated with poor adherence to immunosuppressive medication (49). Non-compliance to medications can dangerously affect the outcomes of kidney transplantation (50, 51) and, together with alcohol consumption and cigarette smoking (52, 53), is a hallmark of depression (54). The detrimental effect of mood disorders on physical health might be also explained by other mechanisms, such as autonomic dysfunction (55), impaired cellular immune response (56), heightened inflammation (57), and increased platelet aggregation (58). Finally, in patients affected by mood disorders, harm could also come from treatments, as there is evidence that antidepressant medication use, which represents the most prescribed drug for mood disorders, is associated with increased mortality and all-cause graft failure in the year following transplantation (59). Even though this could just represent an association, the consumption of other medications used to treat mood disorders, such as antipsychotics or lithium, represents instead a well-established risk for poorer physical health (60, 61). In our study, the presence of mood disorders remained a significant predictor of increased healthcare costs even when excluding the influential point.

Regarding somatization, it was found to be a predictor of higher costs. Compared with the general population, this tendency to experience and communicate somatic distress in response to psychosocial stress (and to seek medical help for it) has been associated with a higher hospital length of stay, higher inpatient costs, and more specialist visits (62). Patients with these conditions often present with vague and difficult-to-identify symptoms, leading to detrimental economic effects (63). In fact, the annual medical costs for “somatizers” have indeed been found to be 2.3 times that for a “non-somatizer', with three times as many hospitalizations (62). Furthermore, KTRs affected by this cluster of syndromes might be more exposed to iatrogenic harm (64–67), leading to a further increase in hospital stays, examinations, and costs.

Furthermore, some limitations of our study should be also mentioned. First, the lack of data regarding the costs of ambulatory care and their changes on the basis of psychosocial clinically significant conditions in KTRs. However, it is complex to quantify the economic burden of this activity as it requires the systematic quantification of the additional costs, which is not always comparable, due to the multiple medical and surgical procedures. Second, the absence of a control group with chronic kidney disease in other settings. Third, some demographics, such as the socioeconomic status (68) (a combined measure of education, income, and occupation) of KTRs, biochemical (69, 70), and ultrasound (71, 72) data were not available to better characterize the population. Additionally, the therapeutic protocols to treat chronic kidney rejection, including steroid dosage, and the economic contribution of physical activity levels, both modifiable risk factors of mental health (73–75), were not evaluated. Finally, no healthcare cost before the kidney transplant was collected.

## 5. Conclusion

This study demonstrated that higher healthcare costs for hospital admissions and emergency department access were strongly predicted by the DCPR diagnosis of somatization cluster and the ICD diagnosis of mood disorder, respectively. In addition, these healthcare costs were associated with a higher risk of poor outcomes until death in kidney transplant recipients. Further studies of cost analysis, cost-effectiveness, cost-benefit, and cost minimization analysis should be conducted to estimate the economic advantages of early diagnosis and treatment of psychosocial syndromes in kidney transplant recipients. Indeed, the healthcare allocation strategy, a pressing question in the transplantation community, should be rethought to invest accurately the resources that are even more limited; therefore, a comprehensive systematic economic analysis of the physical, physiological, and social aspects is needed.

## Data availability statement

The original contributions presented in the study are included in the article/Supplementary material, further inquiries can be directed to the corresponding author.

## Ethics statement

The studies involving human participants were reviewed and approved by the Ethical Committee AVEC. The patients/participants provided their written informed consent to participate in this study.

## Author contributions

Conceptualization: YB. Investigation: LZ and YB. Formal analysis: AP. Data curation: NN and FG. Writing and editing—original draft: YB and LZ. Writing—review and editing: PE and FB. Supervision: RC and AS. Validation: LG. All authors have read and agreed to the published version of the manuscript.
