# Supplementary material for: Psychosocial determinants of healthcare use costs in kidney transplant recipients
Source: Front Public Health. 2023 Jun 2;11:1158387. doi: 10.3389/fpubh.2023.1158387 (PMC10272730; doi:10.3389/fpubh.2023.1158387)

**Supplementary Figure 1**. The diagnostic graphical distribution of residual errors and fitted values used for testing the linear regression model.


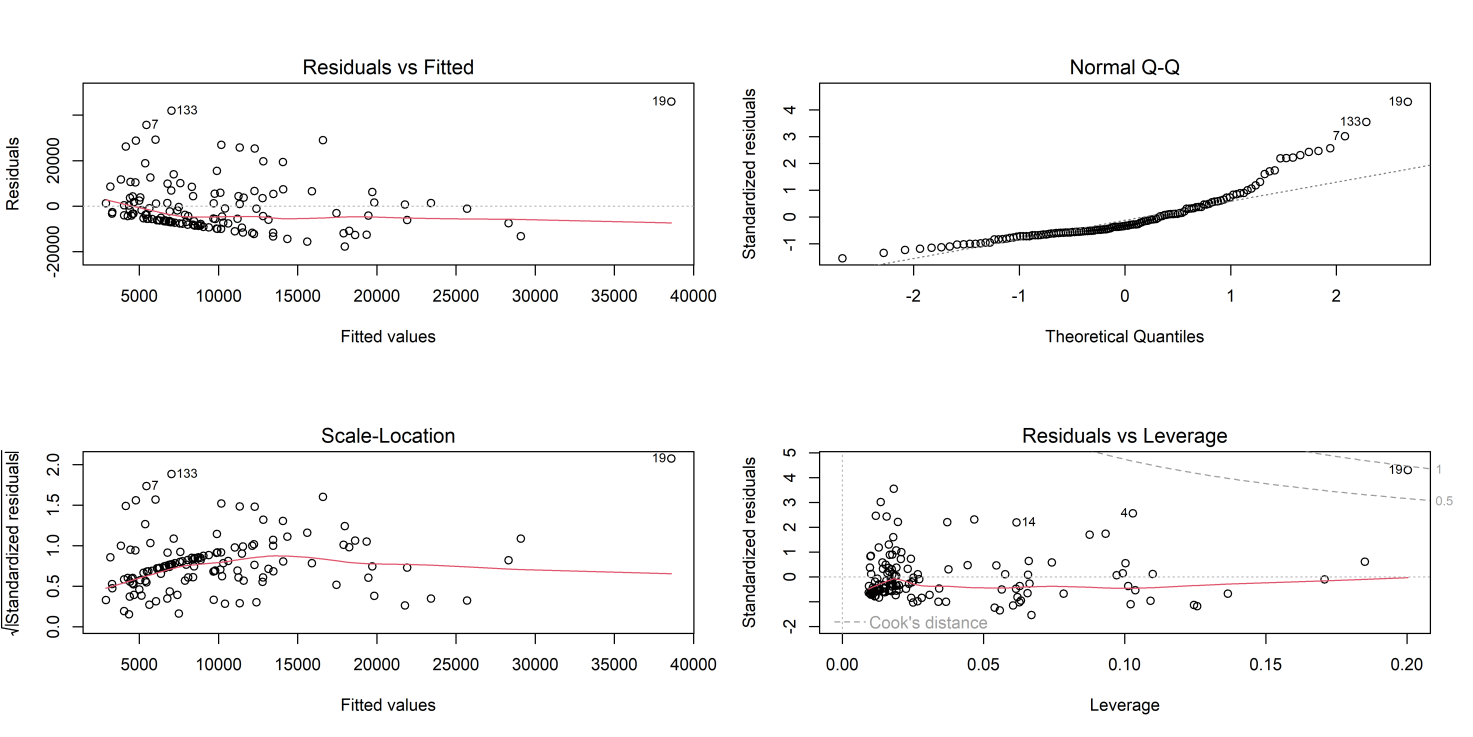

Supplement: Supplementary file 1 [file Data_Sheet_1.docx]
